# Can We Go Online for Sports Injury Prevention? A Systematic Review of English-Language Websites with Exercise-Based Sports Injury Risk Reduction Programmes

**DOI:** 10.1186/s40798-021-00373-z

**Published:** 2021-10-30

**Authors:** Aleksandra Katarzyna Mącznik, Poonam Mehta, Mandeep Kaur

**Affiliations:** 1 Institute for Integrated Sports Medicine, School of Medicine, Keio University, 35 Shinanomachi, Shinjuku-ku, Tokyo, 160-8582 Japan; 2 Graduate School of Health, Discipline of Physiotherapy, University of Technology Sydney, Ultimo, Australia; 3 Department of Kinesiology, University of Virginia, Charlottesville, VA USA

**Keywords:** Sports injury risk reduction, Sports injury prevention, Sports injuries, Exercise programme, Online health resources

## Abstract

**Background:**

Preventing sports injuries is at the forefront of sports medicine. Although effective preventive strategies in scientific literature exist, their implementation is lagging behind. The Internet could support the translation of knowledge from the literature to end-users, but the quality of the online resources would have to be assured. This online-based systematic review is to assess availability, readability, quality, and content of the websites presenting exercise-based sports injury risk reduction (prevention) programmes. Moreover, the quality of reporting and contents of the exercise programmes were assessed.

**Methods:**

Google, Yahoo, and Bing were searched on 2 September 2018. We used ‘sports injury prevention program*’ and ‘sports injury prevention warm-up’ as search phrases. The owners/authors of the included websites were asked for further recommendations on online resources. Search updates were run in DuckDuckGo on 15 May 2020 and 22 August 2021. Eligible websites were active, in English, and contained instructions for the exercise/s aiming at sports injury prevention. Two reviewers independently screened the links and previews and performed an in-depth appraisal of included websites. The website quality was assessed using JAMA framework criteria and Health on the Net Foundation Code of Conduct (HONcode) certification. The readability of websites was assessed using the Flesch-Kincaid Reading Ease score. The reporting appraisal of exercise programmes was done using the modified Consensus on Exercise Reporting Template (CERT).

**Results:**

Among 480 websites screened, 16 were eligible with an additional four recommended and nine found in search updates (29 in total). None of the websites was certified by HONcode. The overall quality of websites was low 2.1 ± 1.0/4, but overall readability was high 67 ± 17/100. The average quality of reporting of exercise programmes was low 5.79 ± 3.1/12. Websites with community input had the lowest readability, but the highest quality, and vice versa websites run by businesses had the highest readability, but the lowest quality. Eight websites presented programmes tested for effectiveness.

**Conclusions:**

Overall, the quality of the websites was low, but their readability was high. Improvements required are relatively easy to implement (i.e. including the date when the website was updated, applying for HONcode certification) and extremely important (e.g. providing resources on which the website’s content is based). There are some sports injury risk reduction programmes reported with high quality and effectiveness-tested available online for team sports, but none for individual sports.

*Trial Registration* This review has been registered in the PROSPERO (CRD42019107104).

## Key Points

- This review has identified 29sports injury risk reduction exercise programmes on the Internet.
- The quality of websites presenting these programmes was low in general, but their overall readability was high.
- There are a few well-reported effectiveness-tested exercise programmes online available for team sports, but none for individual sports.

## Introduction

Considerable incidences of sports injuries are reported worldwide [1], and their consequences affect an athlete’s life in both the short and long term including costs, pain, and medical complications to name just a few [2, 3]. Therefore, injury risk reduction is of primary importance. Sports injury prevention efforts may aim at multiple factors such as changing the competition rules, improving sports equipment, or the way facilities are prepared for competition [4, 5]. These efforts also include strategies focused on the neuromuscular preparedness of an athlete. Effective exercise-based programmes targeting the optimization of neuromuscular performance have been developed and published in the scientific literature, but this availability did not directly translate to their implementation in sports [6–9].

The lag in implementation may be related to the dissemination of the programmes mainly being done through scientific publications. This may not be an optimal way to deliver exercise programmes to the end-users such as athletes, coaches, trainers, clinicians, or parents. Multiple aspects influence limitations of scientific publications, and these include: publishers often posing access restrictions on the articles; the scientific language of research articles; specialist vocabulary used in articles as they are targeted for the audience of researchers, rather than athletes, coaches, trainers or parents; limited figures and videos; and word count restrictions.

The Internet’s ubiquity and low entry barrier allows for easy dissemination of health-related information online [10]. Online injury prevention resources may contribute to a wider distribution of injury risk reduction programmes and their subsequent implementation [11]. On the Internet, there are no restrictions on the format, the number of words, figures or diagrams used, and videos are relatively easy to implement. These aspects offer unique possibilities to disseminate injury prevention programmes in a rich and engaging way. However, as anyone can freely publish on the Internet, the online resources are prone to two main issues, namely low trustworthiness [12] related to the inexistent quality control, and hindered visibility of good resources among the vast noise. Therefore, before the online resources can be adopted successfully, they need to go through a process of quality assessment [13, 14].

There are already third-party initiatives to assess quality specifically in online health information such as the Health on the Net Foundation Code of Conduct (HONcode) initiative [15]. The HONcode initiative aims at certifying websites that fulfil eight quality criteria (authoritative, complementarity, privacy, attribution, justifiability, transparency, financial disclosure, and advertising policy). The difficulty of the HONcode’s process is that the authors or owners have to apply for their website to be assessed. Therefore, an alternative assessment, independent from the authors’ request, needs to be performed.

Apart from issues with quality appraisal, the online resources face a problem of the low visibility of high-quality sources among the low-quality majority of the websites. As the Internet is vast, it may be overwhelming and tedious to find what one needs [16]. Clinicians and others supporting athletes’ health and performance need targeted resources. There is a need for creating a dedicated website/database aggregating different resources for injury prevention in sports along with their appraisal.

The primary purpose of this review was to assess the availability and appraise the readability, quality, and content of the websites presenting exercise-based sports injury risk reduction programmes. Another purpose of the review was to assess the quality of reporting and content of exercise programmes presented on included websites. Finally, we aggregated the results on a publicly accessible website with filtering functionality.

## Methods

### Protocol Registration

This online-based systematic review has been registered in the PROSPERO under the number CRD42019107104 (https://www.crd.york.ac.uk/prospero/display_record.php?RecordID=107104) and reported using the PRISMA checklist [17]. A detailed protocol of this review was also published elsewhere [18].

### Information Sources and Search Strategy

The three search engines: Google, Yahoo, and Bing were searched on 2 September 2018. The search phrases were developed from the literature, and pilot tested. We used ‘sports injury prevention program*’ as a phrase that would be likely used by a person who looks for this kind of information online. The phrase ‘sports injury prevention warm-up’ was also used as a variation of a previous phrase. The second phrase contains ‘warm-up’ as many sports injury risk reduction exercises are incorporated in, or replace, the warm-up. We have also asked all the owners/authors of the included websites for recommendations on additional online resources they were aware of.

On 15 May 2020 and 22 August 2021, we (AKM) updated the search. The same search terms and search strategy were used with one exception: we have used a different search engine. As the project developed, the authors learned about the DuckDuckGo search engine (DuckDuckGo.com). This search engine does not collect personal data and therefore allowed for searching independently of location or influence of usual searches.

### Website Eligibility

The website was considered eligible if it was active, in English (chosen for its status as a dominant language on the Internet), and included instructions (in a form of text, picture and/or video) for the exercise/s aiming at sports injury prevention (risk reduction). No restrictions were applied for injury type, sex, or age of the target population.

The website was excluded if it was inactive, in other languages than English, or expired between the search day (2 September 2018) and the end of the data extraction process (22 August 2021).

### Website Selection

Two reviewers (PM and AKM) independently screened through the links. Then, the reviewers went through a preview of the website, and only if it was deemed eligible, the whole website was inspected in depth. If there were discrepancies in the eligibility assessment, the third reviewer (MK) was consulted. A discussion was carried out until a consensus was reached within the research team.

### Websites’ Appraisal

#### Website’s Quality

The quality of each website was assessed independently by two reviewers (PM and MK) using two measures. The JAMA framework criteria [19] were used to assess if authors’ credentials are present (*authorship*), references and sources are listed (*attribution*), ownership, sponsorship, and advertising are disclosed (*disclosure*) and update date is provided (*currency*). Also, HONcode [15] certification was checked for.

#### Website’s Readability

The readability of websites was assessed using the Flesch–Kincaid Reading Ease (FKRE) score (https://www.webfx.com/tools/read-able/). This measures the reading ease, with a score from 0 to 100. A low score suggests the text is complicated to understand, and a score between 60 and 80 suggests the text is easy to read by a 12- to 15-year-old [20, 21]. Additionally, a percentage of complex words was noted along with the age at which the website’s content can be easily comprehended.

#### Website’s Content Assessment

Type of the website was assessed as either academic, commercial, health business, community, news, public education, blog, or other [22, 23]. Also, we noted the country of origin of the website or exercise programme and the presence or absence of advertisements.

### Exercise Programmes Appraisal

The reporting quality of exercise programmes was assessed independently by two reviewers (AKM and MK) using the modified Consensus on Exercise Reporting Template (CERT) [24]. Only the items not possible to assess in online resources were excluded, leaving 12 items (1, 3, 6, 7a, 8, 9, 10, 12, 13, 14a, 14b, and 15).

### Data Analysis and Synthesis

Data for each website and each exercise programme were extracted by two research team members (PM, MK) and checked by the third researcher (AKM). Website characteristics were described using frequencies. Websites’ quality was summarized by mean and SD for all websites found in this review as well as for each type of website. Additionally, frequencies of each JAMA component were calculated with the presence or absence of HONcode certification. Mean and SD for readability of all and each type of website were calculated along with frequencies and percentages of complex words and target age group. Frequencies of exercise programmes characteristics were calculated including programmes’ aim, targeted audience, programme frequency and duration, number and type of exercises used, equipment required, and presence or absence of testing in the scientific study. The quality of reporting of exercise programmes was summarized by mean and SD. Exercise programmes that scored high were summarized descriptively.

## Results

The results of this review are also available on a custom-built website with a filtering functionality (https://healthylivingscience.com/projects/online-exercise-based-sports-injury-risk-reduction-programmes/).

### Websites’ Characteristics

#### Number and Characteristics of the Websites

**Fig. 1:**
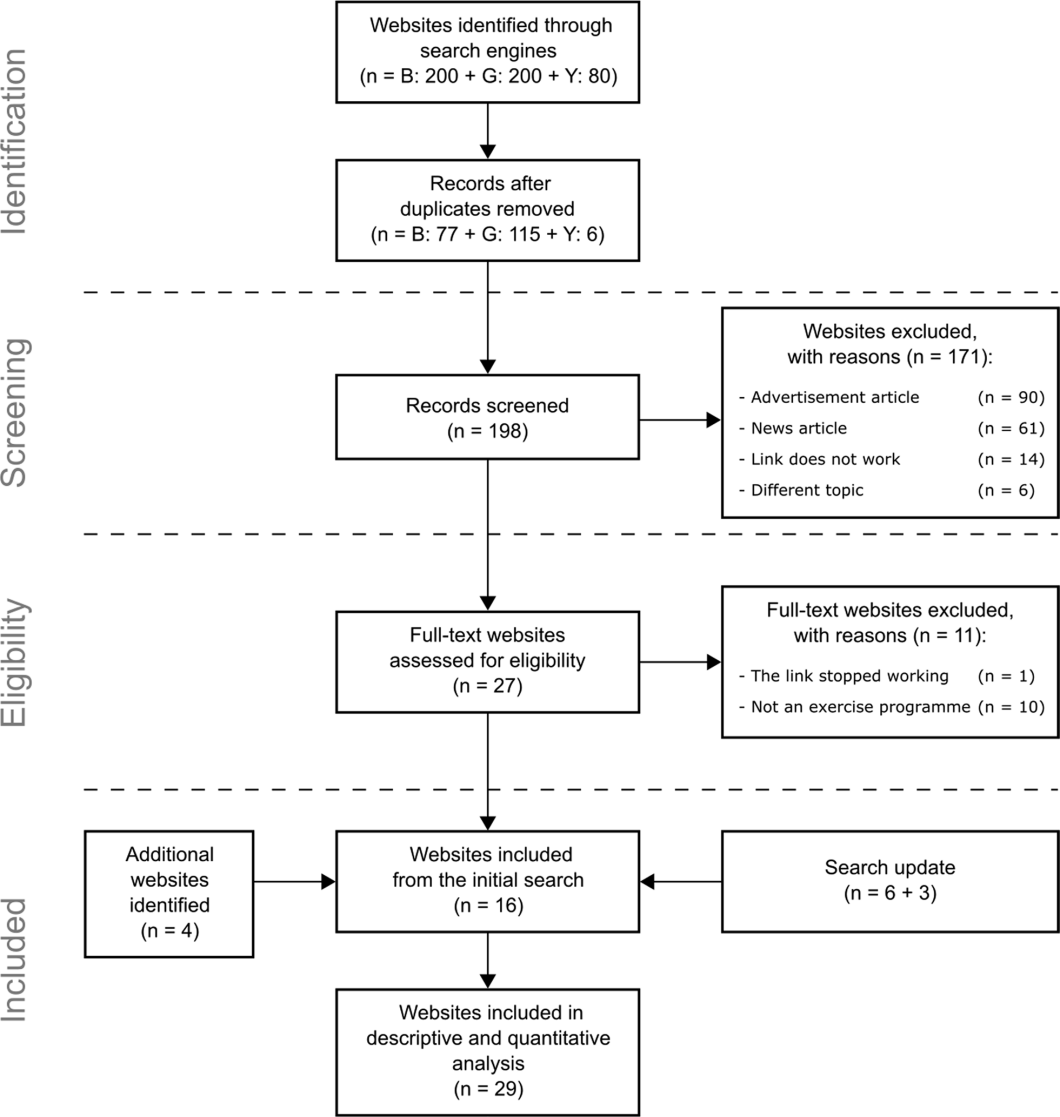
PRISMA flowchart. B—Bing, G—Google, Y—Yahoo

Among 480 links initially retrieved, 16 websites contained sports injury risk reduction programmes (Fig. 1). An additional four websites were referred by the owners/authors of the websites obtained in the search. The updated searches revealed an additional six and three websites, respectively. In total, 29 websites were included in the descriptive and quantitative analysis. The characteristics of the websites are presented in Table 1.

**Table 1:** Characteristics of the websites

Programme	Country of origin	Website’s type	Ads	Readability (0–100)	% of complex words	Easily understood by people aged	Benefits (B), risks (R), precautions (P) listed or discussed*	Pictures or diagrams*	Videos*	JAMA benchmark criteria score (0–4) Total: authorship attribution disclosure currency*	HONcode certification*
1. ACC SportSmart: Coaches’ Kit	New Zealand	Public education	No ads	79.2	9.18	12–13	B y R n P y	y	n	2: n n y y (2002)	n
2. ACL Injury Prevention Program	USA	Health business	Consult ads	64.8	12.02	12–13	B y R n P y	y	n	1: n n y n	n
3. ACL Injury Prevention Programme for the Competitive Female Athlete	USA	Health business	Consult ads	71.4	6.67	12–13	B y R n P y	y	n	2: y n y n	n
4. ACL Prevention Programme by Syracuse Orthopedic Specialists	USA	Health business, Public education	No ads	65.8	6.98	8–9	B n R n P y	y	n	1: n n y n	n
5. Activate Injury Prevention Exercise Programme	UK	Community, public education, academic	Club ads, product ads	47.0	23.88	14–15	B y R n P y	y	y	3: y y y n	n
6. Baseball Pitchers and Throwers Ten Exercise Program	USA	Health business	Consult ads	66.3	13.14	12–13	B y R n P n	y	n	1: n n y n	n
7. FIFA 11 + Injury Prevention Advanced Warmup via wakemed.org	USA, Norway	Health business	Consult ads	65.8	15.67	12–13	B y R n P y	y	y	1: n n y n	n
8. FIFA 11 + Injury Prevention Basic Warm-Up via wakemed.org	USA, Norway	Health business	Consult ads	65.3	16.99	12–13	B y R n P y	y	y	1: n n y n	n
9. Footy First	Australia	Public education, academic, community	Club ads	44.3	17.73	14–15	B y R n P y	y	y	4: y y y y (2011)	n
10. GAA15 for Gaelic Games athletes	Ireland	Public education, academic	No ads	55.8	22.53	12–13	B y R n P y	y	y	2: y n y n	n
11. Hamstring Injury Prevention	USA	Health business	Consult ads	58.1	11.65	14–15	B n R n P n	n	y	1: n n y n	n
12. Injury Prevention Routine by Seattle Pediatric Sports Medicine	USA	Health business, public education	No ads	80.3	4.16	12–13	B n R n P y	y	y	1: n n y n	n
13. Injury Prevention Workout for Basketball via stack.com	USA	Public education	Product ads	61.8	13.63	13–14	B y R n P n	n	n	2: y n n y (2012)	n
14. KIPP (Knee Injury Prevention Program)	USA	Health business	Consult ads	61.6	15.95	13–14	B y R n P y	n	y	4: y y y y (2006–2007)	n
15. The KNEE Programme Netball	Australia	Public education	Consult ads	44.3	20.21	14–15	B y R n P y	n	y	2: y n y n	n
16. Little League Baseball Injury Prevention Programme by Massachusetts General Hospital Sports Medicine	USA	Health business	No ads	96.7	4.76	6–7	B y R n P y	y	n	2: y n y n	n
17. NetballSmart by Netball New Zealand	New Zealand	Public education	Product ads	64.5	12.59	12–13	B y R n P y	y	y	3: n y y y (2019)	n
18. OSTRC Shoulder Injury Prevention Programme for handballers	Norway	Public education, academic	No ads	64.1	19.57	13–14	B y R n P n	n	y	4: y y y y (2017)	n
19. PEP (Prevent Injury, Enhance Performance)	USA	Academic, health business	Consult ads	73.2	14.08	11–12	B y R n P y	y (for stretching only)	n	1: n n y n	n
20. Professional Physical Therapy Injury Prevention Programme for young soccer players	USA	Health business, public education	Consult ads	43.7	25.67	15–16	B y R n P y	n	y	2: n n y y (2014)	n
21. Sanford Knee Injury Prevention Program	USA	Health business	Consult ads	91.8	6.19	7–8	B y R n P y	y	n	2: n y y n	n
22. Sports Conditioning for the Female Knee: An Injury Prevention Program	USA	Health business	Consult ads	86.1	8.38	10–11	B y R n P y	n	n	3: y y y n	n
23. Sports Injury Prevention by PhysioAdvisor.com	Australia	Health business	Consult ads, product ads, membership ads	23.7	30.85	16–17	B y R n P y	y	n	3: y n y y (2019)	n
24. Sports Physiotherapy Sideline Safety Workbook	New Zealand	Public education	No ads	91.9	4.81	7–8	B y R n P y	y	n	2: y n y n	n
25. Stop sports injuries: An injury Prevention Curriculum for Coaches	USA	Health business, public education	No ads	74.7	5.19	12–13	B y R n P y	n	n	4: y y y y (2011)	n
26. The 11	USA, Norway	Public education	No ads	84.6	6.40	8–9	B y R n P y	y	n	2: y n y n	n
27. Warm-up, Cool-down and Stretch by ACC SportSmart	New Zealand	Public education	No ads	90.5	6.35	7–8	B y R n P y	y	n	1: n y n n	n
28. Warm-up exercises for Field Hockey by JYPhysiotherapy	UK	Health business	Consult ads	63.3	15.28	13–14	B y R n P n	n	y	2: y y n n	n
29. WIPP programme	USA	Health business	Consult ads, product ads	59.5	19.93	13–14	B y R n P n	y	n	1: n n y n	n

*y yes, *n* no, *JAMA framework* The Journal of the American Medical Association framework, *HONcode* Health on the Net Foundation Code of Conduct

1. http://www.marching.co.nz/assets/PDFS/00-results/coacheskit.pdf

2. https://loptonline.com/acl-injury-prevention-program/

3. http://www.swansonmcarthurpt.com/aclprogram_11_-rysc.pdf

4. https://www.sosbones.com/media/2126/acl-prevention-program.pdf

5. https://www.englandrugby.com/participation/coaching/activate

6. https://www.upmc.com/services/sports-medicine/for-athletes/baseball/education-material/throwers-ten-exercise-program

7. https://www.wakemed.org/ncfc-injury-prevention-warmup

8. https://www.wakemed.org/ncfc-improve-your-power-performance

9. http://www.aflcommunityclub.com.au/fileadmin/user_upload/Coach_AFL/Become_a_Coach/Accreditation/FootyFirst_-_Manual.pdf

10. https://learning.gaa.ie/Gaelic15

11. https://www.childrenshospitalvanderbilt.org/patient-education/hamstring-injury-prevention

12. https://seattlepediatricsportsmedicine.com/injury-prevention/injury-prevention-warm-up-program/injury-prevention-routine/

13. https://www.stack.com/a/injury-prevention-workout-for-basketball

14. http://kipp.instituteforsportsmedicine.org/

15. https://knee.netball.com.au/about/

16. http://sudburybaseball.com/2e5ca83d-a7fd-45d1-95f7-8735947a7429/Text/Documents/1398/16034.pdf

17. http://www.netballnz.co.nz/useful-info/Netball-smart?fbclid=IwAR3kvttG0Q6hSMBdCttUBaHzR9HB57c9PmV1SnqbObZHFbHBaVnOl4p7LAw

18. https://www.facebook.com/EISphysiotherapy/videos/516355868708829/

19. https://health.usf.edu/medicine/orthopaedic/smart/pep

20. https://www.professionalpt.com/services/injury-prevention-for-young-athletes/

21. https://www.sanfordpower.com/wp-content/uploads/2017/04/014000-00428-BOOKLET-Skipp-Exercises-8_5x8_5-1.pdf

22. https://www.massgeneral.org/ortho-sports-medicine/conditions-treatments/pdfs/Sports-Conditioning-for-the-Female-knee.pdf

23. https://www.physioadvisor.com.au/health/injury-prevention/

24. https://www.sportsphysiotherapy.org.nz/sideline%20management.pdf

25. https://www.stopsportsinjuries.org/STOP/Downloads/Resources/CoachesCurriculumToolkit.pdf

26. https://www.kort.com/uploadedFiles/KORT/Content/Services/Sports_Medicine/Concussion_Management/FIFA-the-11-Booklet.pdf

27. http://acceleratephysio.co.nz/wp-content/uploads/2013/07/ACC-Warm-up-cool-down.pdf

28. https://www.jyphysiotherapy.com/hockey/warm-up-exercises-field-hockey/

29. https://sportsmetrics.org/training/wipp/

The websites (or programmes) originated from counties such as Australia (#9, 15, 23), Ireland (#10), New Zealand (#1, 17, 24, 27), Norway (#7–8, 18, 26), UK (#5, 28), and USA (#2–4, 6–8, 11–14, 16, 19–22, 25–26, 29). The types of websites were: health business or practitioner (*n* = 18; #2–4, 6–8, 11–12, 14, 16, 19–23, 25, 28–29), community (*n* = 2; #5, 9), public education/portal (*n* = 15; #1, 4–5, 9–10, 12–13, 15, 17–18, 20, 24–27), and academic (*n* = 5; #5, 9–10, 18–19). None of the retrieved websites was classified as commercial, news, blog, or other.

In general, the websites were accessible for free, with one exception which had a paid membership option (#23) allowing to access more content. Most of the websites contained advertising (*n* = 19): sports club ads (*n* = 2; #5, 9), appointment (consult) with health professional, clinic or hospital ads (*n* = 15; #2–3, 6–8, 11, 14–15, 19–23, 28–29), or product ads (*n* = 5; #5, 13, 17, 23, 29).

Regarding the presentation of the exercise programmes, most of the websites listed the benefits and/or precautions (*n* = 28; #1–10, 12–29) of using the presented programme. However, none of the programmes have listed potential risks.

In terms of media presence, 20 websites (#1–10, 12, 16–17, 19, 21, 23–24, 26–27, 29) provided pictures or diagrams of exercises and 13 websites (#5, 7–12, 14–15, 17–18, 20, 28) had videos demonstrating how to perform the exercises available.

#### Websites’ Quality

The average quality ± SD of the websites as measured by the JAMA benchmark criteria [19] was 2.1 ± 1.0 out of 4, ranging from 1 (*n* = 10) to 4 (*n* = 4). Most websites (*n* = 26; #1–12, 14–26, 29) disclosed an ownership, sponsorship, and advertising (disclosure), 15 websites (#3, 5, 9–10, 13–16, 18, 22–26, 28) reported the authors’ credentials (authorship), but only ten (#5, 9, 14, 17–18, 21–22, 25, 27–28) listed references or sources (attribution) and nine (#1, 9, 13–14, 17–18, 20, 23, 25) provided the date of the last update (currency). Only three websites (#17–18, 23) were updated recently (in 2017 or 2019); update for the other six (#1, 9, 13–14, 20, 25) ranged from 2002 to 2014.

Comparing the quality (as measured by JAMA benchmark criteria [19]) of different types of websites, the websites produced by a health business (*n* = 18; #2–4, 6–8, 11–12, 14, 16, 19–23, 25, 28–29) had on average the lowest quality 1.83 ± 1.0. Public education websites (*n* = 15; #1, 4–5, 9–10, 12–13, 15, 17–18, 20, 24–27) scored 2.33 ± 1.0, and academic websites (*n* = 5; #5, 9–10, 18–19) scored 2.80 ± 1.3. The highest quality was presented by websites with community input (*n* = 2; #5, 9) 3.50 ± 0.7. Quality of the websites for which more than one stakeholder contributed to the content (*n* = 9; #4–5, 9–10, 12, 18–20, 25) was higher with an average score of 2.33 ± 1.4, and the quality of the websites with single stakeholder input (*n* = 19; #1–3, 6–8, 11, 13–17, 21–24, 26–29) was lower averaging at 2.00 ± 0.9. None of the websites was certified by HONcode [15], nor was in the process of obtaining a certification.

#### Websites’ Readability

The websites assessed in this review scored on average 67 ± 17 out of 100 on the FKRE [20, 21], ranging from 24 (hard to read) to 97 (very easy to read). Eleven websites (#1, 3–4, 12, 16, 21–22, 24–27) used less than 10% of complex words, with the highest percentage of complex words exceeding 30% (#23). In summary, six websites (#4, 16, 21, 24, 26–27) were suitable for children’s level of literacy, 20 (#1–3, 5–15, 17–19, 22, 25, 28–29) for adolescents’ level, and only two (#20, 23) for adults.

Comparing the readability of different types of websites, the websites produced by a health business (*n* = 18) scored on average the highest on FKRE 67 ± 17. Public education websites (*n* = 15) scored 66 ± 17, and academic websites (*n* = 5) scored 57 ± 12. The lowest readability score was presented by websites with community input (*n* = 2) 46 ± 2. The readability of the websites with contributions from more than one stakeholder (*n* = 9) was lower with an average score of 61 ± 14, and the readability of the websites with single stakeholder input (*n* = 19) was higher, averaging at 70 ± 19.

#### Exercise Programmes’ Characteristics

Programmes’ characteristics are summarized in Table 2. Nine programmes (#1, 12, 19, 20, 23–25, 27, 29) were aimed at the prevention of sports injuries in general and ten at some type of lower limb injury (#2–4, 9–11, 14–15, 21–22). Sixteen programmes were designed to reduce injuries in specific sports: athletics (#21), Australian football (#9), baseball (#6, 16), basketball (#13), Gaelic games (#10), handball (#18), hockey (#28), netball (#15, 17), rugby (#5), or soccer (#2–3, 7–8, 20, 26). No programme was designed specifically for individual (as opposed to team) sports.

**Table 2:** Exercise-based sports injury risk reduction programmes’ characteristics

Programme	Focus of the programme	Sport level*	Age group* Sex/gender	Frequency per week* Session duration*	Programme duration (weeks or # of sessions)*	Type of exercise	Number of exercises	Progression*	Setting	Equipment	Additional components*	Evidence of effectiveness Result*	Quality via modified CERT (0–12)
1. ACC SportSmart: Coaches’ Kit	Sports injury	NM	NM NM	3–5 times 20–60 min	NM	Warm-up, stretching, running, strength, balance, jumping	> 24	Some rules provided	Individual	Wall	Hydration, nutrition, protective equipment, fair play, screening, injury management,training load management	NM	8
2. ACL Injury Prevention Program	ACL injury	Soccer	NM F	2–3 times; during the season 15 min	NM	Warm-up, stretching, strengthening, plyometrics, and sport specific agility training, cool down	24	NM	Individual	Not needed	NM	NM But based on PEP [28]	1
3. ACL Injury Prevention Programme for the Competitive Female Athlete	ACL injury	Soccer	Adolescent F	2–3 times 15 min	NM	warm-up, stretching, strengthening, plyometrics, agility	24	Rules for under 11, some exercises have progressions	Individual	Cone	NM	NM But based on PEP [28]	8
4. ACL Prevention Programme by Syracuse Orthopedic Specialists	ACL injury	NM	NM NM	NM NM	NM	Jumping, lunging, strength, single-leg skill practice	10	NM	Individual	Chair, ball	NM	NM	2
5. Activate Injury Prevention Exercise Programme	Rugby injuries including concussion	Rugby Levels for Under 15, under 16, under 17–18, adults	Undue 15, undue 16, undue 17–18, adults M	3 times 15–20 min	6–8 (age groups) or 4–6 (adults) weeks e per phase	Running, Core strength, balance, agility, jumping	12 (age groups)—15 (adult) exercises per phase	Under 15, under 16, under 17–18, adult versions, 4 (age groups)-7 (adults) progressive phases	Individual, in pairs	Partner	NM	Y Effective [23, 24]	10
6. Baseball Pitchers and Throwers Ten Exercise Program	Throwing injuries	Baseball, American football, other throwing sports	NM NM	NM NM	NM	Strength	18	Some exercises have progressions	Individual	Band, wall, weights	NM	NM	5
7. FIFA 11 + Injury Prevention Advanced Warmup via wakemed.org	Soccer injuries	Soccer	NM NM	At least 2 times 20 min	3 stages (beginner, intermediate, advanced), 4 weeks each	Strength, plyometrics, balance	6	Beginner, intermediate and advanced programme	Individual, in pairs	Ball, partner	NM	Y Effective [25]	9
8. FIFA 11 + Injury Prevention Basic Warm-Up via wakemed.org	Soccer injuries	Soccer	NM NM	At least 2 times 20 min: 8-min running, 10-min strength, plyometric, balance	24 sessions	Running, strength, balance, plyometric, core stability	15	Progressions available in 20b	Individual, in pairs	Partner, ball, cones	NM	Y Effective [25]	9
9. Footy First	Leg injuries	Community Australian football	NM NM	At least 2 times 20 min	17 + weeks	Running, Strengthening, balance, landing and side-stepping skills	12 warm-up, 5 level 1–5 exercises	Programme divided into 5 levels, also some exercises are progressed	Individual, in pairs	Partner, 2 balls, 4 cones	NM	NM	12
10. GAA15 for Gaelic Games athletes	Lower limb injuries	Hurling, Gaelic football	Under-18 and adults NM	2 times 15 min	Start of each training	Running, strengthening, balance and controlled partner contacts, jumping, hamstrings, sport-specific exercises in moderate and high speed	19	Some exercises have levels	Individual, in pairs	Partner, box, weights	NM	Y effective	8
11. Hamstring Injury Prevention	Hamstring strain	NM	NM NM	NM NM	NM	Strength	1	NM	In pairs	Partner, mat	Signs & symptoms of a hamstring injury	NM	4
12. Injury Prevention Routine by Seattle Pediatric Sports Medicine	Sports injuries	NM	NM NM	NM NM	NM	Dynamic mobility, strength, motor control, agility	19: 7 dynamic mobility, 5 strength, 3 motor control, 4 agility	Some of the exercises are progressed by an increase in reps	Individual, in pairs	Partner	NM	NM	2
13. Injury Prevention Workout for Basketball via stack.com	Basketball injuries	Basketball	NM NM	NM NM	NM	Dynamic warm-up, general movement, stretching, basketball-specific exercises, mobility sequence, jumping	Around 40 exercises	NM	Individual	Foam roller, band	Warm-up barefoot, foam rolling	NM	0
14. KIPP (Knee Injury Prevention Program)	Knee injuries, lower limb injuries	NM (but tested on soccer and basketball athletes)	Adolescent F	2–3 times 15 min	NM	Strength, plyometric, balance, coordination and agility, active stretching	17 essential exercises, 12 game day exercises, 6 practice exercises	NM	Individual	Not needed	Hydration, increase in training load < 10%/week, protective equipment, pre-season physical assessment, ACL injury epidemiology, risk factors & mechanisms	Y Effective in female soccer and basketball athletes [26]	6
15. The KNEE Programme Netball	Knee injuries	Netball, junior (under 14), recreational, elite	11–14 junior, recreational (14 +), elite (state and national level) NM	At least 2 times (2–3 times) 10–12 min	All year round	Running, jumping, strength, balance/landing, agility	17 (junior), 21 (recreational), 23 (elite) but many replacement exercises	Some exercises are progressed by adding ball or variative in throws of the ball	Individual, in pairs	Ball, partner	Technique development	NM	7
16. Little League Baseball Injury Prevention Programme by Massachusetts General Hospital Sports Medicine	Baseball injuries	Baseball	Little league age players NM	3 times NM	NM	Strength, power, endurance of the shoulder complex	10 exercises	NM	Individual	Band, weights, bench	NM	NM	1
17. NetballSmart by Netball New Zealand	Netball injuries	Netball	NM NM	2–3 times 15–20 min	NM	Strengthening, running, dynamic preparation, netball specific preparation	17 (with many replacement exercises)	Some exercises are progressed	Individual, in pairs	Cones, ball, partner	Hydration, recovery, roller recovery, nutrition, sleep	NM	11
18. OSTRC Shoulder Injury Prevention Programme for handballers	Shoulder injuries	Handball	Elite F & M	NM NM	NM	Strength	NM	NM	Individual, in pairs	Partner, bands	NM	Y Effective in elite female and male handballers [27]	5
19. PEP (Prevent Injury, Enhance Performance)	Sports injuries and performance	NM	NM F	NM 15–20 min	NM	Warm-up, stretching, strengthening, plyometrics, and sport specific agilities	Warm-up: 3 Stretching: 6 Strengthening: 3 Plyometrics: 5 Agility: 3 Alternative exercises: 5	Progression by adding repetitions, or unstable surface	Individual	Ball, cone	NM	Y Effective (non-randomized design) [28]	7
20. Professional Physical Therapy Injury Prevention Programme for young soccer players	Sports injuries	Soccer, youth	Youth F, M	At least 3 times per week 25–35 min	NM	Dynamic warm-up, proprioception, plyometrics, strength, cool down	Dynamic warm-up: 13 Proprioception: 3 Plyometrics: 5 Strength: 7 Cool down: 7	Some exercises have progression	Individual	Partner, ball, band, foam roller, cones	Stretching, nutrition, hydration, functional training, conditioning	NM	8
21. Sanford Knee Injury Prevention Program	Knee injuries	Athletics Middle school, high school, collegiate, professional	NM NM	2–3 times per week 15–20 min	At least 8–12 weeks	Dynamic stretching, hip muscle activation, strengthening, plyometrics, agilities	29	NM	Individual	Band	NM	NM	5
22. Sports Conditioning for the Female Knee: An Injury Prevention Program	Knee injuries	NM	NM F	NM 15–20 min	6 sessions	Active warm-up, stretching, strengthening, plyometric, agility	14–21	Yes, from session to session	Individual	Medicine ball, hurdles, ladder	NM	NM But based on PEP [28]	7
23. Sports Injury Prevention by PhysioAdvisor.com	Sports injuries	NM	NM NM	1–7 times 5–15 min	NM	Core stability, balance, proprioceptive, pelvic stability exercises, scapular stability exercises	7 core, 3 balance, 3 pilates, 3 scapular exercises	Basic, intermediate, advanced exercises	Individual	Mat, Swiss ball	Warm-up, nutrition, hydration, technique, environment conditions	NM	5
24. Sports Physiotherapy Sideline Safety Workbook	Sports injuries	NM	NM NM	NM 5–10 min or more	NM	Warm-up, dynamic stretching, cool down	8 dynamic stretching	Some exercises have progressions	Individual	Rail to hold on to	Protective equipment, footwear, environment – surface, climate, technique (skill), fair play	NM	4
25. Stop sports injuries: An injury Prevention Curriculum for Coaches	Sports injuries including overuse injuries, concussion, heat illness	NM	Youth NM	NM NM	NM	Plyometrics, running, lunges	> 13 exercises	NM	Individual, in pairs	Not needed	Training load < 10% increase, heat & cold, dehydration conditions symptoms & management, pre-season conditioning	NM	3
26. The 11	Soccer injuries and performance	Soccer	NM NM	Every training session after warm-up 10–15 min	NM	Core stabilization, eccentric training of the thigh muscles, proprioceptive training, dynamic stabilization, plyometrics	10	Some exercises (e.g. “perform slowly at first, but once you feel more comfortable, speed it up”)	Individual, in pairs	Ball, partner	Fair play	Y No effect [25]	7
27. Warm-up, Cool-down and Stretch by ACC SportSmart	Sports injuries	NM	NM NM	NM 5–15 min	NM	Running, jumping, stretching, sport-specific exercises, e.g. short sprints, shuttle runs, changing direction, shooting drills	3 aerobic, 12 stetching,6 sport-specific	NM	Individual	Ball, wall	NM	NM	2
28. Warm-up exercises for Field Hockey by JYPhysiotherapy	Hockey injury	Field hockey	NM NM	2 times NM	NM	Warm-up, stretching, strengthening, plyometrics, agility, balance	24	NM	Individual	Box, foam, roller	NM	NM But based on FIFA11 + [25]	6
29. WIPP programme	Sports injuries	NM	NM NM	NM 20 min	Preseason and in-season	Dynamic warm-up, jumps, strength, agility	17	Some exercises have progression	Individual	Wall, band	NM	NM	6

*CERT* consensus on exercise reporting template, *NM* not mentioned, *Y* yes, *M* male, *F* female

1. http://www.marching.co.nz/assets/PDFS/00-results/coacheskit.pdf

2. https://loptonline.com/acl-injury-prevention-program/

3. http://www.swansonmcarthurpt.com/aclprogram_11_-rysc.pdf

4. https://www.sosbones.com/media/2126/acl-prevention-program.pdf

5. https://www.englandrugby.com/participation/coaching/activate

6. https://www.upmc.com/services/sports-medicine/for-athletes/baseball/education-material/throwers-ten-exercise-program

7. https://www.wakemed.org/ncfc-injury-prevention-warmup

8. https://www.wakemed.org/ncfc-improve-your-power-performance

9. http://www.aflcommunityclub.com.au/fileadmin/user_upload/Coach_AFL/Become_a_Coach/Accreditation/FootyFirst_-_Manual.pdf

10. https://learning.gaa.ie/Gaelic15

11. https://www.childrenshospitalvanderbilt.org/patient-education/hamstring-injury-prevention

12. https://seattlepediatricsportsmedicine.com/injury-prevention/injury-prevention-warm-up-program/injury-prevention-routine/

13. https://www.stack.com/a/injury-prevention-workout-for-basketball

14. http://kipp.instituteforsportsmedicine.org/

15. https://knee.netball.com.au/about/

16. http://sudburybaseball.com/2e5ca83d-a7fd-45d1-95f7-8735947a7429/Text/Documents/1398/16034.pdf

17. http://www.netballnz.co.nz/useful-info/Netball-smart?fbclid=IwAR3kvttG0Q6hSMBdCttUBaHzR9HB57c9PmV1SnqbObZHFbHBaVnOl4p7LAw

18. https://www.facebook.com/EISphysiotherapy/videos/516355868708829/

19. https://health.usf.edu/medicine/orthopaedic/smart/pep

20. https://www.professionalpt.com/services/injury-prevention-for-young-athletes/

21. https://www.sanfordpower.com/wp-content/uploads/2017/04/014000-00428-BOOKLET-Skipp-Exercises-8_5x8_5-1.pdf

22. https://www.massgeneral.org/ortho-sports-medicine/conditions-treatments/pdfs/Sports-Conditioning-for-the-Female-knee.pdf

23. https://www.physioadvisor.com.au/health/injury-prevention/

24. https://www.sportsphysiotherapy.org.nz/sideline%20management.pdf

25. https://www.stopsportsinjuries.org/STOP/Downloads/Resources/CoachesCurriculumToolkit.pdf

26.https://www.kort.com/uploadedFiles/KORT/Content/Services/Sports_Medicine/Concussion_Management/FIFA-the-11-Booklet.pdf

27. http://acceleratephysio.co.nz/wp-content/uploads/2013/07/ACC-Warm-up-cool-down.pdf

28. https://www.jyphysiotherapy.com/hockey/warm-up-exercises-field-hockey/

29. https://sportsmetrics.org/training/wipp/

Seven programmes (#2–3, 14, 18–20, 22) were specifically designed for female athletes, three for male athletes (#5, 18, 20), and the rest did not specify the sex or gender. Two programmes (#16, 25) were specifically designed for children (below 10 years of age), six (#3, 5, 10, 14, 15, 20) for adolescents (10–19 years of age), four (#5, 10, 15, 18) for adults, and 20 did not specify the targeted age. All websites found in this review focused on primary prevention. The level of sport participation was specified only by five programmes, but each one of them used a different naming system: U15 (under 15 years of age), U16, U17–18, and adults (#5); under 18 and adults (#10); junior, recreational, and elite (#15); middle school, high school, collegiate, professional (#21), or community level (#9).

Seventeen programmes advised to perform injury prevention exercises at least twice a week (#1–3, 5, 7–10, 14–17, 20–21, 23, 26, 28), and 12 programmes (#4, 6, 11–13, 18–19, 22, 24–25, 27, 29) did not specify the frequency. Nine programmes (#2–3, 10, 14–15, 23–24, 26–27) were short (up to 15 min), nine programmes (#5, 7–9, 17, 19, 21–22, 29) lasted between 15 and 20 min, and eight (#4, 6, 11–13, 16, 25, 28) did not specify the session duration. There were three programmes (#5, 7, 22) designed for four weeks, eight programmes (#5, 7–10, 15, 21, 29) designed to be performed for more than four weeks, and 20 programmes (#1–4, 6, 11–14, 16–20, 23–28) did not specify the programme duration.

Regarding the type of exercise, all programmes but three (#6, 11, 18) used multiple types of exercises. Most programmes used strengthening (n = 24; #1–12, 14–22, 28–29) and plyometrics (*n* = 12; #2–3, 7–8, 14, 19–22, 25–26, 28) or jumping (*n* = 8; # 4–5, 10, 13, 15, 27, 29). Other types of exercises included running (n = 9; #2, 5, 8–10, 15, 17, 25, 27), agility (n = 11; #2–3, 5, 12, 14–15, 19, 21–22, 28–29), balance (n = 10; #1, 5, 7–10, 14–15, 23, 28), stretching (*n* = 11; #1–3, 13–14, 19, 21–22, 24, 27–28), sport-specific exercises (*n* = 6; #2, 4, 10, 17, 19, 27), proprioceptive training (*n* = 3; #20, 22, 25), and landing training (*n* = 2; #9, 15). Most programmes (*n* = 19; #2–13, 15–17, 20–22, 28) focused mainly on lower limb exercises, three programmes focused on full body exercises (#1, 18–19), and one focused on upper body exercises (#14).

The number of exercises ranged from one exercise to 40. Six programmes (#4, 7, 11, 15, 24, 26) had below 10 exercises, 11 programmes (#5–6, 8–10, 14, 16, 22–23, 25, 29) had between 11 and 20 exercises, and 13 programmes (#1–3, 12–15, 19–22, 27–28) had more than 20 exercises. Eighteen programmes (#1, 3, 5–10, 13, 15, 17, 19–20, 22–24, 26, 29) had some exercise progressions available. Most of the programmes (*n* = 28; #1–10, 12–29) could be performed by an individual, and 12 programmes (#5, 7–12, 15, 17–18, 25–26) had exercises to be performed in pairs.

Only three programmes (#2, 14, 25) did not require any equipment, and the rest of the programmes required some basic sports equipment such as a ball (*n* = 12; #4, 7–9, 15, 17, 19–20, 22–23, 26–27) or a band (n = 7; #6, 13, 16, 18, 20–21, 28). Most of the programmes (*n* = 18; #2–10, 12, 16, 18–19, 21–22, 27–29) did not have any additional components. However, seven programmes addressed other aspects related to the risk of an injury such as hydration (#1, 14, 17, 20, 23, 25), nutrition (#1, 17, 20, 23), training load management (#1, 14, 25), climate/environment (#1, 23, 24–25), protective equipment (#1, 14, 24), and/or sleep (#17). Additionally, four programmes (#1, 11, 13, 25) included information on sports injuries (mechanism, treatment), three promoted rolling (#13, 17, 28), and three promoted (#1, 24, 26) fair play.

### Exercise Programmes’ Reporting Appraisal

The average reporting of exercise programmes was scored 5.79 ± 3.1 out of 12 (on modified CERT) [24] and ranged from 0 to 12. The quality of reporting of 16 programmes (#2, 4, 6, 11–14, 16, 18, 21, 23–25, 27–29) was assessed as low (scored ≤ 6 on modified CERT) and 13 (#1, 3, 5, 7–10, 15, 17, 19–20, 22, 26) as high (scored 7–12). There were two main issues in reporting: 1) the majority of the programmes were lacking information on how the exercise is tailored to the individual and 2) decision rule on the level the exercises should be started at (items 14b and 15 on the CERT). The most represented sports in online prevention were soccer (*n* = 6; #2–3, 7–8, 20, 26), baseball (*n* = 2; #6, 16) and netball (*n* = 2; #15, 17). The average reporting quality for programmes in each of the sports was therefore calculated at 7.0 (ranging from 1 to 9) for soccer, 3 (1–5) for baseball, and 9 (ranging from 7 to 11) for netball.

Eight programmes were tested for effectiveness in sports populations: seven (#5 [25, 26], 7–8 [27], 10, 14 [28], 18 [29], 19 [30]) were effective and one was not (#26 [27]). An additional four programmes (#2–3, 22, 28) were based on tested (and effective) programmes [27, 30], and 18 programmes (#1, 4, 6, 9, 11–13, 15–17, 20–21, 23–25, 27, 29) were neither tested nor based on tested programmes.

A detailed analysis of *exercise programmes reported with high quality* is presented below. For soccer, out of six programmes (#2–3, 7–8, 20, 26), five (#3, 7–8, 20, 26) were of high quality of reporting on CERT [24], but out of these, only two (#7–8) were proven effective (two versions of FIFA 11+) [27]. None of the programmes designed for baseball (#6, 16) were reported with high quality. The *KNEE programme Netball* from Netball Australia (#15) and Netball Smart from Netball New Zealand (#17) were of high reporting quality and with additional videos for all the exercises available. Netball Smart also presented advice on additional aspects of risk reduction such as optimal hydration, nutrition, sleep, and recovery practices. These two programmes did not provide any information about being tested for effectiveness though. The next programme of high reporting quality, and additionally tested with a positive outcome [25, 26], was *Activate Injury Prevention Programme* for rugby (#5). The *Footy First* designed for Australian football (#9) was of high reporting quality, also having videos and images available, but not tested. *GAA15* (#10) for Gaelic Games athletes was reported with high quality and tested, but the reference to the study was not presented. The *Prevent Injury, Enhance Performance* (PEP) Programme (#19) was reported with high quality and tested with positive results in female soccer players (non-randomized design of the study) [30]. The last two programmes reported with high quality were *Sports Conditioning for the Female Knee: An Injury Prevention Programme* (#22) and ACL Injury Prevention Programme for the Competitive Female Athlete (#3). Both programmes were based on the PEP Programme (#19) that was tested [30], and has shown effectiveness.

## Discussion

The quality of found websites varied with most of them scoring low on the JAMA benchmark criteria. Additionally, none of the websites had HONcode certification. Readability assessment, on the other hand, has shown encouraging results with most websites being written in plain English. In terms of the quality of reporting of exercise programmes, there are some high-quality programmes available online for team sports, but none for individual sports. Additionally, the results of this review have been publicized on a purposively built website with filtering functionality (https://healthylivingscience.com/projects/online-exercise-based-sports-injury-risk-reduction-programmes/) to allow for easy access to both reviewed websites and their appraisal.

### Websites’ Quality

The low quality of websites appraised in this study is an important issue, and a part of a bigger problem with the quality of health-related information online [31, 32]. The use of the Internet as a source of health-related information is growing, but the quality of the content is not [32]. Social media are used for obtaining health-related advice by half of its users, with 18% of these users never verifying the information they receive [33]. Ninety percent of those who verify the information use basic Google search [33]. In the injury prevention context, 32% of basketball coaches are sourcing their injury prevention knowledge from the Internet; the only more popular resources are other coaches [11]. Without the high-quality resources freely available online, it may be difficult to achieve the goal of reducing injuries in sport.

The quality of websites presenting exercise-based injury programmes can be improved. Lack of date of the last update for most of the websites (currency) was the main issue in websites’ quality when assessed against JAMA benchmark criteria. Also, out of the websites that reported the date of an update, only three were up to date (others were dated 6–19 years previously). Fortunately, this aspect of the website’s quality can be easily fixed by the website’s authors and owners. Lack of website’s update date may influence users’ perception of trustworthiness [34], and the lack of currency poses a question about the accuracy of evidence being presented.

Looking at the type of the website in relation to its quality, those produced with community involvement were of the highest quality. However, there were only two websites of this type, so any conclusions should be drawn with caution. Websites classified as health businesses have shown the lowest average quality. This may be due to the main aim of these websites being to recruit clients or promote a product rather than disseminate health-related information. Having input from multiple (more than one) stakeholder/s appears to improve the overall quality of the website too. This result aligns with the literature suggesting that multiple stakeholder consultations are a crucial stage in knowledge translation developments [35, 36].

None of the websites was certified by HONcode. HONcode is an initiative attempting to standardise the reliability of medical and health information available online. A recent study by Daraz and colleagues [31] has shown that only 18% of online health information is certified by HONcode, and another study by Hanley and collegues [37] has found that 11% of websites on autopsy for the general public were HONcode certified. The lack of certification may, therefore, mean that sports injury prevention exercise programmes are not considered ‘medical and health information’ yet and therefore the owners/authors may not feel the need to attempt certifying them. To pass HONcode certification, the website has to satisfy certain criteria; satisfying these criteria may help to improve the overall quality and content of the website, and therefore can be advised.

### Websites’ Readability

The readability of the websites also varied, but the overall outcome of the assessment is positive. The average readability of the websites is at a plain English readability level [20, 21, 38]. Six websites were written at a level understood by children and other 20 by adolescents. This result is not surprising considering that children would not be expected to run sports injury risk reduction exercise programmes by themselves, but rather be guided by adults. On the other hand, having most of the programmes understandable by adolescents under the age of 15 years is encouraging. Youth can comprehend the consequences of sports injuries and the importance of preparation and risk reduction, can follow instructions, and therefore can potentially perform the injury prevention programme by themselves.

More active involvement of youth in injury prevention could potentially help coaches/health professionals in implementation. Thirty to forty percent of coaches [7, 39] claimed time restriction is a barrier toinjury prevention programme implementation in adolescents. Therefore, if youth could support or even run the injury prevention programme by themselves, it could be part of the solution to the implementation crisis we observe.

### Trustworthiness of Websites

The low quality and high readability of websites found in this review may result in mixed impressions and therefore affect the websites’ perceived trustworthiness [34]. Trust in a website is a prerequisite for users to implement the potential exercise programme in their training.

The fact that websites published by businesses had the highest readability, but the lowest quality, and community websites had the highest quality, but the lowest readability, may pose a problem. The user may overestimate the trustworthiness of business websites because they are easy to read, but not trust the community websites due to lower readability. Ideally, we would like to see high-quality websites also have high readability to send a clear message about trustworthiness and reduce barriers to implementation.

Additionally, most of the websites found in this review contain advertising. The high number and distracting nature of advertising and banners on a website can negatively affect the credibility of that site [40]. Finally, some of the exercise programmes were presented on external websites rather than on the websites affiliated with the authors. An example is FIFA 11+ (#7–8), which appeared on a website not branded as FIFA's.

How the above-mentioned aspects influence the perception of trustworthiness of these websites, and how this will affect exercise programme implementation is unclear and requires further investigation. In the meantime, to allow for easier dissemination of online sports injury prevention programmes, the provision of external assessment contributed by this review may be of help.

### Exercise Programmes’ Reporting Appraisal

The low quality of reporting of the exercise programmes is concerning. Some of the programmes did not specify information regarding required frequency per week, session duration, or programme duration. Low quality of reporting will probably limit their usefulness for direct use by young athletes or their parents. However, these programmes may be still used by professionals with additional knowledge, such as sports clinicians or coaches. Low-quality-reported programmes had mainly two issues in reporting: the majority lacked information on how the exercise is tailored to the individual or on the decision rule for the starting level. Both issues require expertise to allow the adoption of the presented programme to suit a particular sport or individual [41].

Eleven exercise programmes were reported with high quality (by CERT), and out of these, six were tested and found effective, and these should be considered as best candidates for immediate implementation. This shows that in the English-speaking Internet, there is a handful of both effective and well-reported exercise-based injury prevention programmes that could be potentially implemented. Bridging this gap will require substantial efforts as developing effective exercise programmes and producing high-quality online resources doubles the challenges.

### Potential for Implementation of the Exercise Programmes

The implementation of exercise-based injury risk reduction programmes found in this review will obviously depend on the empirical evidence for their effectiveness as discussed above. However, there are other aspects that can influence implementation.

The strength of many of the online programmes found in this review is their heavy use of pictures, diagrams, and videos. This is an excellent utilization of the Internet medium, as the use of pictures and videos may facilitate comprehension of exercises, improve their understanding and accuracy in performance, and allow for easier implementation. Visual presentation of the Internet-based injury prevention resources can be leveraged also to promote them for example with the use of social media. Additionally, most of the programmes could be performed individually, or in pairs, with minimal equipment use which enhances their potential for easy implementation.

There are, however, some issues that may hinder the implementation of online programmes. Overall, the programmes found in this review aimed at preventing sports injuries in general, with only a few designed for specific team sports or sex/gender. As risk factors for injuries differ from sport to sport and between males and females, these general programmes may not be an optimal way to change the landscape of rising sports injuries, especially as most of them were not tested for effectiveness. Also, within the same sport and the same sex, optimal injury reduction programmes are likely to differ depending on the targeted injury. If no targeted injury is specified, it may be harder to elicit an initial buy-in from coaches, athletes, and/or their parents as general sports injury may not be a “good enough” reason to invest in running an injury prevention programme.

None of the websites mentioned the risks of performing an exercise programme. This is an important omission and requires immediate improvement. The readability level of most of the websites allows them to be used by young people rather than always be guided by a professional or another adult. However, without additional information, young people may struggle with fully understanding the context of the exercises they perform. A previous study [42] has indicated that players and staff members in professional soccer teams strongly support the use of evidence-based injury prevention exercise protocols. However, perceptions vary considerably between teams regarding which exercises can prevent injuries, who holds the responsibility for injury prevention, and when preventive exercises should be performed. This indicates that authors of websites may want to consider adding information on the occurrence of injuries, risk factors leading to injuries, and the role of an individual player and a team in injury prevention.

The multifactorial nature of sports injuries is hardly ever addressed on the included websites. Admittedly, this review included the websites hosting the exercise sports injury prevention programmes, which usually focus on addressing intrinsic risk factors related to athletes’ neuromuscular preparedness. However, the causes of sports injuries are multifactorial [43, 44], and therefore, presentation of strategies to address other risk factors could be included alongside the exercises. Although mainly focused on exercise alone, a few websites used the opportunity to address other aspects of prevention such as sleep, hydration, nutrition or training load, and climate conditions’ management. Would it be better to have websites addressing multiple risk factors or would it be too overwhelming for the end-user? How both solutions affect the usability of these resources is unclear and requires investigation in future studies.

### Limitations

This study has several limitations. Firstly, there are some other online exercise-based sports injury risk reduction programmes not detected by the search strategy. This resulted both from inherent difficulties posed by popular search engines (e.g. IP geography or search history) and often lack of contact details (or response) of the owners of the websites. For example, we found Netball Smart, but not Rugby Smart. We hope that in the next iteration of this review, we will be able to include more websites using a more comprehensive search strategy. Secondly, as there were only 29 websites in total, some of the categories of analyses performed had low representation which may have skewed the results. As the number of resources will increase with time, this problem should be reduced. Thirdly, only websites in the English language were searched for and included in this review and therefore the review may have missed programmes in languages other than English. Lastly, efforts to optimize systematic appraisal of online resources are still in their infancy [45]. As the authors of this review, we have tried to follow standard guidelines for a ‘systematic review’, but admit the need for the development of a framework adapted to conduct reviews of online resources in the future.

## Conclusions

This review appraised 29 websites containing exercise-based sports injury risk reduction programmes. Overall, the quality of the websites was low, but their readability was high. Improvements required are relatively easy to implement (i.e. including the date when the website was updated, applying for HONcode certification) and extremely important (e.g. providing resources on which the website’s content is based). In terms of the quality of reporting of exercise programmes themselves, there are some sports injury risk reduction programmes reported with high quality and tested for effectiveness available online for team sports, but none for individual sports.

## Data Availability

All data are presented in this manuscript.

## Abbreviations

CERT: Consensus on Exercise Reporting Template
FKRE: Flesch–Kincaid Reading Ease
HONcode: Health on the Net Foundation Code of Conduct

## References

[1] Birrer RB, O'Connor FG, Kane SF (2016). Musculoskeletal and sports medicine for the primary care practitioner.

[2] Maffulli N, Longo UG, Gougoulias N, Caine D, Denaro V (2010). Sport injuries: a review of outcomes. Br Med Bull.

[3] Maffulli N, Longo UG, Gougoulias N, Loppini M, Denaro V (2010). Long-term health outcomes of youth sports injuries. Br J Sports Med.

[4] Blauwet C, Webborn N, Kissick J, Lexell J, Stomphorst J, van de Vliet P, Lazarovski D, Derman W (2019). When van Mechelen's sequence of injury prevention model requires pragmatic and accelerated action: the case of para alpine skiing in Pyeong Chang 2018. Br J Sports Med.

[5] Vriend I, Gouttebarge V, Finch CF, Van Mechelen W, Verhagen EA (2017). Intervention strategies used in sport injury prevention studies: a systematic review identifying studies applying the Haddon matrix. Sports Med.

[6] Hanson D, Allegrante JP, Sleet DA, Finch CF (2014). Research alone is not sufficient to prevent sports injury. Br J Sports Med.

[7] Norcross MF, Johnson ST, Bovbjerg VE, Koester MC, Hoffman MA (2016). Factors influencing high school coaches’ adoption of injury prevention programs. J Sci Med Sport.

[8] O'Brien J, Donaldson A, Finch CF (2016). It will take more than an existing exercise programme to prevent injury. Br J Sports Med.

[9] Verhagen E, Voogt N, Bruinsma A, Finch CF (2014). A knowledge transfer scheme to bridge the gap between science and practice: an integration of existing research frameworks into a tool for practice. Br J Sports Med.

[10] Winker MA, Flanagin A, Chi-Lum B, White J, Andrews K, Kennett RL, DeAngelis CD, Musacchio RA (2000). Guidelines for medical and health information sites on the internet: principles governing AMA web sites. JAMA.

[11] Räisänen AM, Owoeye OBA, Befus K, van den Berg C, Pasanen K, Emery CA (2021). Warm-ups and coaches' perceptions: searching for clues to improve injury prevention in youth basketball. Front Sports Act Living.

[12] Dutta-Bergman M (2003). Trusted online sources of health information: differences in demographics, health beliefs, and health-information orientation. J Med Internet Res.

[13] Beaunoyer E, Arsenault M, Lomanowska AM, Guitton MJ (2017). Understanding online health information: evaluation, tools, and strategies. Patient Educ Couns.

[14] Duffy M (2000). The Internet as a research and dissemination resource. Health Promot Int.

[15] Boyer C, Baujard V, Geissbühler A (2011). Evolution of Health Web certification through the HONcode experience. Stud Health Technol Inform.

[16] Ybarra ML, Suman M (2006). Help seeking behavior and the Internet: a national survey. Int J Med Inform.

[17] Moher D, Liberati A, Tetzlaff J, Altman DG, The PRISMA Group (2009). Preferred reporting items for systematic reviews and meta-analyses: the PRISMA statement. PLoS Med.

[18] Mącznik AK, Mehta P, Kaur M (2019). Online exercise-based sports injury risk reduction programs—a ‘systematic review’ protocol. Phys Ther Rev.

[19] Silberg WM, Lundberg GD, Musacchio RA (1997). Assessing, controlling, and assuring the quality of medical information on the Internet: Caveant lector et viewor—let the reader and viewer beware. JAMA.

[20] Cotugna N, Vickery CE, Carpenter-Haefele KM (2005). Evaluation of literacy level of patient education pages in health-related journals. J Community Health.

[21] Fowler GE, Baker DM, Lee MJ, Brown SR (2017). A systematic review of online resources to support patient decision-making for full-thickness rectal prolapse surgery. Tech Coloproctol.

[22] Charnock D, Shepperd S, Needham G, Gann R (1999). DISCERN: an instrument for judging the quality of written consumer health information on treatment choices. J Epidemiol Community Health.

[23] Charvet-Berard AI, Chopard P, Perneger TV (2008). Measuring quality of patient information documents with an expanded EQIP scale. Patient Educ Couns.

[24] Slade SC, Dionne CE, Underwood M (2016). Consensus on exercise reporting template (CERT): modified Delphi study. Phys Ther.

[25] Attwood MJ, Roberts SP, Trewartha G, England ME, Stokes KA (2018). Efficacy of a movement control injury prevention programme in adult men’s community rugby union: a cluster randomised controlled trial. Br J Sports Med.

[26] Hislop MD, Stokes KA, Williams S, McKay CD, England ME, Kemp SP, Trewartha G (2017). Reducing musculoskeletal injury and concussion risk in schoolboy rugby players with a pre-activity movement control exercise programme: a cluster randomised controlled trial. Br J Sports Med.

[27] Thorborg K, Krommes KK, Esteve E, Clausen MB, Bartels EM, Rathleff MS (2017). Effect of specific exercise-based football injury prevention programmes on the overall injury rate in football: a systematic review and meta-analysis of the FIFA 11 and 11+ programmes. Br J Sports Med.

[28] LaBella CR, Huxford MR, Grissom J, Kim KY, Peng J, Christoffel KK (2011). Effect of neuromuscular warm-up on injuries in female soccer and basketball athletes in urban public high schools: cluster randomized controlled trial. Arch Pediat Adolesc Med.

[29] Andersson SH, Bahr R, Clarsen B, Myklebust G (2017). Preventing overuse shoulder injuries among throwing athletes: a cluster-randomised controlled trial in 660 elite handball players. Br J Sports Med.

[30] Mandelbaum BR, Silvers HJ, Watanabe DS, Knarr JF, Thomas SD, Griffin LY (2005). Effectiveness of a neuromuscular and proprioceptive training program in preventing anterior cruciate ligament injuries in female athletes: 2-year follow-up. Am J Sports Med.

[31] Daraz L, Morrow AS, Ponce OJ, Beuschel B, Farah MH, Katabi A, Alsawas M, Majzoub AM, Benkhadra R, Seisa MO, Ding JF (2019). Can patients trust online health information? A meta-narrative systematic review addressing the quality of health information on the Internet. J Gen Intern Med.

[32] Finney Rutten LJ, Blake KD, Greenberg-Worisek AJ, Allen SV, Moser RP, Hesse BW (2019). Online health information seeking among US adults: measuring progress toward a healthy people 2020 objective. Public Health Rep.

[33] Iftikhar R, Abaalkhail B (2017). Health-seeking influence reflected by online health-related messages received on social media: cross-sectional survey. J Med Internet Res.

[34] Scharrer L, Stadtler M, Bromme R (2019). Judging scientific information: does source evaluation prevent the seductive effect of text easiness?. Learn Instr.

[35] Graham ID, Logan J, Harrison MB, Straus SE, Tetroe J, Caswell W, Robinson N (2006). Lost in knowledge translation: time for a map?. J Contin Educ Health Prof.

[36] Richmond SA, McKay CD, Emery CA (2014). Knowledge translation in sport injury prevention research: an example in youth ice hockey in Canada. Br J Sports Med.

[37] Hanley B, Brown P, O’Neill S, Osborn M (2019). Assessment of the quality and readability of online information on autopsy for the general public: a cross-sectional analysis. BMJ Open.

[38] San Giorgi MR, de Groot OS, Dikkers FG (2017). Quality and readability assessment of websites related to recurrent respiratory papillomatosis. Laryngoscope.

[39] Joy EA, Taylor JR, Novak MA, Chen M, Fink BP, Porucznik CA (2013). Factors influencing the implementation of anterior cruciate ligament injury prevention strategies by girls soccer coaches. J Strength Cond Res.

[40] Eysenbach G, Köhler C (2002). How do consumers search for and appraise health information on the world wide web? Qualitative study using focus groups, usability tests, and in-depth interviews. BMJ.

[41] Creighton DW, Shrier I, Shultz R, Meeuwisse WH, Matheson GO (2010). Return-to-Play in Sport: A Decision-based Model. Clin J Sport Med.

[42] O'Brien J, Finch CF (2017). Injury prevention exercise programs for professional soccer: understanding the perceptions of the end-users. Clin J Sport Med.

[43] Huebner BJ, Plisky PJ, Kiesel KB, Schwartzkopf-Phifer K (2019). Can injury risk category be changed in athletes? An analysis of an injury prevention system. Int J Sports Phys Ther.

[44] Mugele H, Plummer A, Steffen K, Stoll J, Mayer F, Müller J (2018). General versus sports-specific injury prevention programs in athletes. PLoS ONE.

[45] Stansfield C, Dickson K, Bangpan M (2016). Exploring issues in the conduct of website searching and other online sources for systematic reviews: how can we be systematic?. Syst Rev.

